# DRACMA one year after: Which changes have occurred in diagnosis and treatment of CMA in Italy?

**DOI:** 10.1186/1824-7288-37-53

**Published:** 2011-11-10

**Authors:** Alessandro Fiocchi, Holger Schunemann, Luigi Terracciano, Marco Albarini, Alberto Martelli, Massimo Landi, Enrico Compalati, Giorgio Walter Canonica

**Affiliations:** 1 Paediatric Division, Department of Child and Maternal Medicine, University of Milan Medical School at the Melloni Hospital, (Via M. Melloni 52), Milan, (20129), Italy; 2 Department of Clinical Epidemiology & Biostatistics, McMaster University Health Sciences Centre, (1280 Main Street West), Hamilton, (ON L8S 4K1), Canada; 3 Italian Federation of Paediatric Medicine, Territorial Paediatric Primary Care Group (C.so Traiano 64/14), Turin, (10135), Italy; 4 Allergy & Respiratory Diseases Clinic, Department of Internal Medicine, University of Genoa, (L.go R. Benzi, 10), Genoa, (16132), Italy

## Introduction

In 2008 the World Allergy Organization (WAO) verified that the existing guidelines on CMA were usually national position papers reflecting local views and needs, with flexible, sometimes evidence-based, strategies [1-4]. Then, a global guideline for IgE-mediated CMA from diagnosis to treatment was developed using the GRADE approach [5]. We review here the first steps of Diagnosis and Rationale for Action Against Cow's Milk Allergy (DRACMA) together with the changes in diagnostic and therapeutic behavior generated by the new guideline.

### How GRADE methodology impact on food allergy guidelines

Challenges with the guideline development process include difficulties in synthesizing evidence on diagnostic tests and therapeutic indications, reconciliation of information obtained through different statistical methods, transparency in evaluation of diagnostic and therapeutic tools, adherence to the clinical questions to which a physician is confronted in real-life [6].

In order to meet these needs, two panels were constituted in DRACMA: a clinical panel and a methodological GRADE revision panel [7]. Three systematic reviews addressing the clinical questions were developed by the GRADE revision panel: 1-diagnosis, 2-use of substitute formulas and 3-immunotherapy for CMA. The GRADE evidence profiles for the clinical questions were developed on these systematic reviews. Summaries of evidence were reviewed by the panel members whose suggestions were incorporated. The quality of the evidence was classified as "high", "moderate", "low " or "very low" [6,8-12]. Finally, the DRACMA guideline panel reviewed the evidence summaries and formulated "strong" or "conditional and/or weak" recommendations. The statements on the underlying values, preferences and remarks are integral parts of the recommendations, and serve to facilitate their accurate interpretation.

### DRACMA implementation

DRACMA was first introduced at the 2009 Buenos Aires World Allergy Congress and in a dedicated Meeting in Milan in February 2010 [13]. After the first publication in WAO Journal in April 2010, DRACMA was replicated in an indexed journal [14]. The worldwide situation in diagnosis and treatment of CMA before DRACMA was described in a round table at the Milan Meeting [15]. The subsequently published NIAID guidelines [16] widely referred to DRACMA, now cited by dozens. Of note, the method used has been indicated as an example of transparency in the development of guidelines and it has become a cornerstone for GRADE methodology [7,8,17-19]. A DRACMA implementation committee has been appointed at WAO in order to favour the diffusion of the guideline. Under its auspices, a GLORIA Module on DRACMA is being developed, national translations are being prepared and a dedicated page on WAO website is in preparation. From this, mobile and I-Pad applications developed by a volunteer copyrighted to WAO will be freely available.

### CMA diagnosis

The DRACMA guidelines provide indications on CMA diagnosis using traditional sensitization tests (SPT and specific IgE determination) vs. the gold standard test (OFC). This allows personalized decision making procedures tailored to the single patient's medical history. As an example, the diagnostic procedure will be different for a child with a recent episode of anaphylaxis and for a child with atopic eczema possibly triggered by cow's milk. The clinical history drives the risk assessment through an estimate of the pretest probability of CMA. Pretest probability can be low, average (most cases) or high. The DRACMA guidelines *always recommend OFC for diagnosing CMA as a strong recommendation *to avoid:

- the risk of anaphylactic reactions at home in false negative sensitization tests,

- unnecessary treatment for false positive cases

- inappropriate resource utilization.

**Figure 1:**
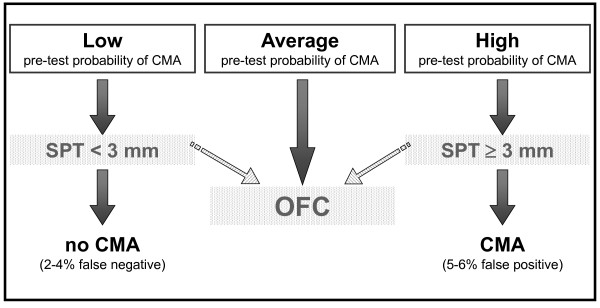
**In settings where OFC is not considered a requirement, should skin prick tests be used for the diagnosis of IgE-mediated cow's milk allergy?**.

**Figure 2:**
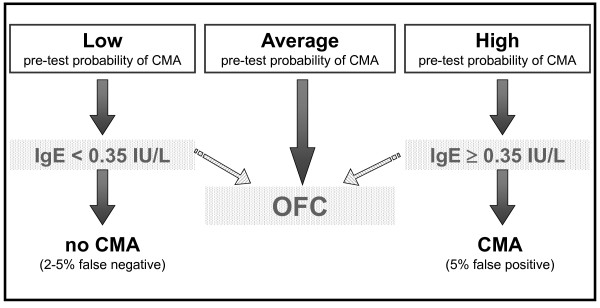
**In settings where OFC is not considered a requirement, should cow's milk-specific immunoglobulin E test be used for the diagnosis of IgE-mediated cow's milk allergy?**.

**Figure 3:**
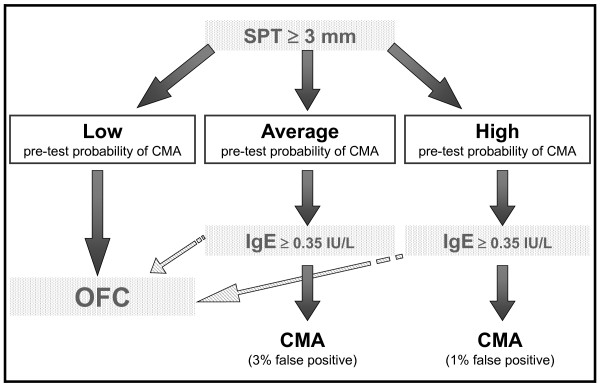
**In settings where OFC is not considered a requirement, should in vitro specific IgE determination be used for the diagnosis of CMA in patients suspected of CMA and a positive result of a skin prick test?**.

**Figure 4:**
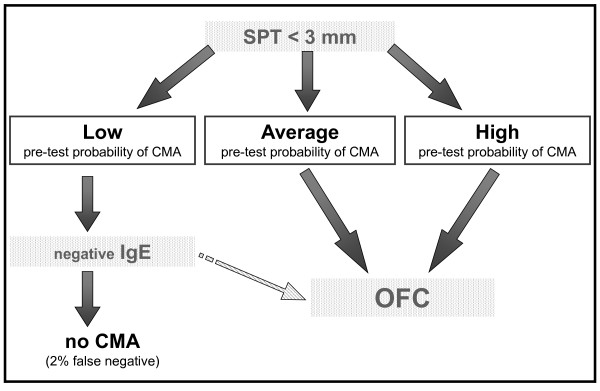
**In settings where OFC is not considered a requirement, should in vitro specific IgE determination be used for the diagnosis of CMA in patients suspected of CMA and a negative result of a skin prick test?**.

However, many reasons (availability of medical and nursing staff, hospitals resources, ability of families to reach reference centers, etc.) may make difficult performing an OFC at the outset. Thus, in selected cases, using a pretest probability estimate can offer an *almost *certain diagnosis simply by performing an SPT and/or specific IgE determination. The diagnosis would be "almost" certain because, as in any decision-making path, there is a small chance of false results. The guidelines indicate the price to pay to avoid performing OFC in all cases and keep it only for selected doubtful cases. The decision to use OFC in all cases (recommendation 1.1 and 2.1) or to recur to sensitization tests in some cases (recommendations 1.2, 1.3, 1.4, 2.2, 2.3, 2.4, 3 and 4) [14] should be taken at local level. Four simple patterns describe these decision processes (Figures 1, 2, 3 and 4). After a scientific review of all the papers available in the literature via the GRADE method, the DRACMA guidelines identified the cut off values for SPT and specific IgE determination results as 3 mm for wheal diameter in the SPT and 0.35 IU/L specific IgE to cow's milk using the CAP-RAST or FEIA method.

### How DRACMA changes diagnostic attitudes in CMA?

The DRACMA recommendations substantially modify the current practice in diagnosing CMA. Per their dictate, the diagnostic OFC performed under the supervision of a specialist remain the best diagnostic strategy, to be performed whenever available. This is so evident that in case OFC is performed out of a research setting, sensitization tests may not be necessary (recommendation 1.1). This is counterintuitive for pediatric allergists, as correct diagnosis of CMA, starting with a suspicion and ending with the OFC, traditionally passes through sensitization tests including SPT, atopy patch tests, and specific IgE determination. Despite the recommendations, it is current practice that these tests base clinical decisions. Thus, very often OFC is not part of the diagnostic workup and is only indicated after an elimination period of a few months or upon a specialist's advice in more severe cases. This exposes whole populations to overdiagnosis of CMA and to excessive use of elimination diets [20,21]. However, there are whole regions in the world and even whole States in the USA where performing an OFC is impossible for practical reasons [15]. In this case, DRACMA recommendations indicate that challenge may not be necessary in many cases. OFCs remain necessary in all cases of high uncertainty. This is also a revolutionary step in CMA diagnosis. The search for a replacement tests has been very active in the past years, a sort of philosopher's stone to avoid OFC whose practice is considered risky, resource- and time-consuming [22]. Specific IgE cut-off points, SPT diameters and/or APT have been proposed as replacement tests. In DRACMA, the limits of these diagnostic practices are clearly indicated and their possible use is re-evaluated. Atopy patch test is not considered useful in the diagnosis of IgE-mediated CMA and molecular diagnosis may be useful, but further data are necessary [23].

### Treatment with CMA replacement formulas

In its therapeutic guidelines DRACMA recommended a milk-free diet for cases of IgE-mediated CMA and the use of appropriate alternative formulas up to at least 2 years of age to meet the nutritional needs of these very young children (Table 1). The complete recommendation set is available at http://www.worldallergy.org/publications/WAO_DRACMA_guidelines.pdf. According to these recommendations, when a substitute formula is needed a milk-based extensively hydrolyzed formula (eHF) is the first choice except in case of anaphylaxis or eosinophilic esophagitis, where AAF is recommended. eHFs should be tested on an outpatient basis before being used at home and new formulas should be monitored for adverse reactions when first administered. eHFs are preferred over soy formulas (SF) to avoid untoward reactions to soy. Conversely, eHF is preferred over extensively hydrolyzed rice formula (eHRF) because more commonly available on the market. Where readily available as in Italy, eHRF can adequately replace eHF. Further studies are required to confirm the benefits of rice protein over SFs. Milks from buffalo, ewe, goat, camel, mare or donkey, cannot constitute the treatment of choice for CMA in the developed world. In particular, goat and ewe milks may expose patients to severe reactions due to cross-reactivity with cow's milk. Camel and dromedary milk can be considered as effective substitutes for children over 2 years of age because for their low protein fraction sequence homology with cow milk. Mare's and donkey's milks can also be considered as valid substitutes particularly (but not exclusively) in children with delayed-onset CMA (e.g. AD). These recommendations deeply change the traditional approach to the choice of a substitute formula. As an example, SFe, widely used, are a second-line choice due to possibility of secondary sensitization and to nutritional drawbacks. In any case, SF cannot be considered under 6 months of age

**Table 1:** Reference Guide to the Recommendations [14]

Clinical presentation	1st choice	2nd choice	3rd choice
Anaphylaxis	AAF^+^	eHF^#§^	SF
Acute urticaria or angioedema	eHF^§♭^	AAF^/SF°	
Atopic dermatitis	eHF^§♭^	AAF^/SF°	
Immediate gastrointestinal allergy	eHF^§♭^	AAF^/SF°	
Allergic eosinophilic oesophagitis	AAF		
Gastroesophageal reflux disease (GERD)	eHF^♭^	AAF	
Cow's milk protein-induced enteropathy	eHF^§♭^	AAF	
Food protein-induced enterocolitis syndrome (FPIES)	eHF*	AAF	
CM protein-induced gastroenteritis and proctocolitis	eHF^♭^	AAF	
Severe irritability (colic)	eHF^♭^	AAF	
Constipation	eHF^♭^	AAF	Donkey milk^^
Milk-induced chronic pulmonary disease (Heiner's syndrome) **	AAF^	eHF	SF

^+ ^recommendation 7.1

^♭ ^recommendation 7.2

* if AAF refusal

§ subject to local availability, HRF can be considered instead than eHF (7.4)

# subject to a negative SPT with the specific formula (panel recommendation)

^AAF if a relatively high value on avoiding sensitization by SF and/or a low value on resource expenditure are placed.

°SF if a relatively low value on avoiding sensitization by SF and/or a high value on resource expenditure are placed.

**this suggestion attributes a high value on avoiding exposure to even residual antigenic cow's milk proteins.

^^based on reports from one case series (chapter 15)

^†^given that more than 50% of such children are allergic to soy, a careful clinical evaluation is necessary (panel recommendation)

### Use of OIT in CMA

The DRACMA guidelines recommend that OIT be administered to patients with IgE-mediated CMA only within the context of formal clinical research to avoid serious adverse effects that negatively offset the increased probability of desensitization to milk. However, due to the rapidly growing literature, a new metanalysis will be produced shortly.

## Conclusion

Pediatric medicine is a science, not an art. In a field traditionally open to various interpretations, as CMA, DRACMA guidelines draw a definitive borderline between diagnostic possibilities, and a clear indication in the choice of the appropriate formula. The application of DRACMA recommendations to the Italian reality should favour the diffusion of the correct diagnostic practices in a context very rich of diagnostic facilities [24] but in need of standardization of the procedures [25]. If correctly applied, they should also modify the composition of the special formulae market, avoiding unnecessary treatments.

## List of Abbreviations

AAF: Amino Acid Formula; AD: Atopic dermatitis; CMA: Cow's milk allergy; DRACMA: Diagnosis and Rationale for Action against Cow's Milk Allergy; eHF: extensively Hydrolyzed (Milk) Formula; eHRF: extensively Hydrolyzed Rice Formula; GLORIA: GLObal Resources In Allergy; GRADE: Grading of Recommendations, Assessments, Development, and Evaluation; OFC: Oral Food Challenge; OIT: Oral Immunotherapy; sIgE: Specific IgE; SF: Soy Formula; SPT: Skin prick test; WAO: World Allergy Organzation.

## Competing interests

The authors declare that they have no competing interests.

## Authors' contributions

All authors read and approved the final manuscript.

## Authors' Information

All authors except MA and GWC belong to the DRACMA Implementation Committee, World Allergy Organization. GWC is the immediate past-president of the World Allergy Organisation
